# Long-term outcomes of congenital adrenal hyperplasia due to 21-hydroxylase deficiency: a retrospective study from a tertiary care center in Saudi Arabia

**DOI:** 10.3389/fendo.2025.1512161

**Published:** 2025-04-17

**Authors:** Haneen Aldalaan, Afaf Alsagheir, Nujud Alghamdi, Raghad Alhuthil, Maha Almslam, Mohamed H. Al-Hamed

**Affiliations:** ^1^ Department of Pediatrics, King Saud Medical City, Riyadh, Saudi Arabia; ^2^ Department of Pediatrics, King Faisal Specialist Hospital and Research Centre, Riyadh, Saudi Arabia; ^3^ Department of Nursing Development and Saudization, King Faisal Specialist Hospital and Research Centre, Riyadh, Saudi Arabia; ^4^ Department of Clinical Genomics, Center for Genomic Medicine, King Faisal Specialist Hospital and Research Centre, Riyadh, Saudi Arabia

**Keywords:** long-term outcomes, congenital adrenal hyperplasia, 21-hydroxylase deficiency, Saudi Arabia, glucocorticoid therapy

## Abstract

**Introduction:**

Data on congenital adrenal hyperplasia (CAH) disorders in the Saudi population are limited. This retrospective study assessed the clinical characteristics ofadolescents and adults with 21-hydroxylase CAH alongside the long-term outcomes of chronic glucocorticoid replacement therapy.

**Methods:**

The study was conducted at the King Faisal Specialist Hospital and Research Centre, Riyadh, Saudi Arabia. The subjects included patients (aged ≥ 14 years) with 21-hydroxylase CAH, who attended the endocrine clinic between 2019 and 2021.

**Results:**

The study found that among the 108 patients with 21-hydroxylase deficiency considered, predominantly females (66.67%), with a median age of 21 years (IQR: 18–30), 93.51% had the classic salt-wasting form, while 6.49% had the nonsalt-wasting form. Glucocorticoid therapy for the patients included prednisone (46.3%), hydrocortisone (37.97%), and dexamethasone (12.03%). Short stature was observed in 30% of the patients, while obesity affected 35.19%. Among the females, 58.33% had oligomenorrhea. In addition, testicular adrenal rest tumors (TARTs) were detected in 44.44% of the males. Metabolic issues included high cholesterol in 95.65%, with 17.33% exhibiting prediabetics. Genetic testing identified CYP21A2 mutations in all patients tested.

**Discussion:**

short stature, obesity, and menstrual irregularities are highly prevalent in females, whereas TARTs are common in males. Although metabolic and bone health outcomes are generally favorable, the variability in hormonal control and its associated complications underscores the need for individualized glucocorticoid therapy. Continuous monitoring and improved treatment strategies are essential for optimizing the quality of life of patients with CAH.

## 1 Introduction

Congenital adrenal hyperplasia (CAH) refers to a group of rare genetic disorders affecting 1 in 10,000–20,000 live births worldwide (1–3). It is an autosomal recessive disease that occurs because of a single gene mutation in the enzyme pathway responsible for cortisol biosynthesis (4). Over 95% of all reported CAH cases result from 21-hydroxylase enzyme deficiency (4). This deficiency results in impaired synthesis of glucocorticoid and/or mineralocorticoid, leading to increased secretion of adrenocorticotropic hormone (ACTH), adrenal hyperplasia, and increased production of androgens (5). CAH can present in three different clinical forms according to the residual enzymatic activity and/or degree of aldosterone deficiency, which include classic severe salt-wasting form (with nearly complete deficiency of 21-hydroxylase enzyme activity), simple virilizing form (with less than 5% of 21-hydroxylase enzyme activity) and non-classical late onset milder form (which have 20%–50% of enzyme activity (2, 4–6). The clinical features of CAH are variable according to the severity of enzyme deficiency, ranging from salt wasting crisis and genital ambiguity in the neonatal period to precocious puberty and accelerated growth secondary to androgen excess in childhood (4, 7).

The introduction of glucocorticoid replacement therapy in the 1950s, along with the widespread implementation of newborn metabolic screening programs worldwide, enabled the early diagnosis and treatment of classic CAH, leading to increased life expectancy in this population (8–10). However, long-term complications related to corticosteroid therapy mis-dosing have become an increasingly recognized health issue (8).

While glucocorticoid and mineralocorticoid replacement therapies remain the standard treatments used to replace deficient cortisol levels and to reduce excessive ACTH, thereby preventing the consequent increase in androgen production (2, 8), nonphysiological doses are needed to achieve this goal, with the risk of unfavorable long-term complications (8, 9). Despite the treatment efficacy in increasing survival rates of patients with CAH, high glucocorticoid therapy doses negatively impact growth, bone health, cardiometabolic health, and fertility (2, 10, 11). While CAH management in children has been established with the presence of international guidelines and recommendations, such guidelines for adults remain unclarified (12).

There are few studies reporting the prevalence of CAH in Saudi Arabia (13, 14). A study conducted in Saudi Arabia between January 2019 and April 2022, examining 54,940 newborns in a single tertiary center, revealed a CAH incidence of 1:325 (15). In addition, as with other international newborn screening programs, the program aimed to detect the classic severe form of CAH by measuring the elevated 17-hydroxyprogesterone concentration (16).

This study aimed to assess the clinical characteristics of adolescents and adults with 21-hydroxylase CAH at the King Faisal Specialist Hospital and Research Centre (KFSHRC) and the long-term outcomes of chronic glucocorticoid replacement therapy.

## 2 Methodology

This was a retrospective study of patients with a confirmed diagnosis of CAH due to 21-hydroxylase deficiency, diagnosed through hormonal testing (elevated 17-hydroxyprogesterone in newborn screening or after an ACTH stimulation test >300 nmol/L) and/or genetic testing confirms *CYP21A2* mutation (17). The study subjects included all adult patients above the age of 14 years (who reached their final height and closed their epiphysis) who attended the endocrine clinic between January 2019 and December 2021.

A total of 108 patients met the inclusion criteria and were enrolled in this study (36 males and 72 females). The individual medical files and charts were reviewed. All clinical, biochemical, and genetic data, alongside long-term complications, including short stature, metabolic syndrome, osteopenia, and reproductive complications, were extracted and analyzed.

### 2.1 Clinical data and definitions

Height was expressed as a standard deviation score (SDS). Normal height was defined as height SDS > - 2 while short stature was defined as height SDS ≤ -2 (18), and severe short stature was defined as height SDS ≤ -3 (19). Near Adult Height (NAH) defined as growth velocity of less than 2 cm per year, with left-hand X-rays confirming epiphyseal plate fusion (20). Body mass index (BMI) was assessed in all patients. Obesity was defined as a BMI at or above the 95th percentile for adolescents and a BMI of 30 kg/m² or greater for adults. Overweight was defined as a BMI between the 85th and 95th percentiles for adolescents and a BMI of more than 25 but less than 30 kg/m² for adults (21).

Hypertension was defined as a systolic BP of at least 140 mm Hg or a diastolic BP of at least 90 mm Hg in adults and prehypertensive BP as a systolic BP between 120 and 140 mmHg or a diastolic BP between 80 and 90 mmHg (22). We assessed androstenedione control based on levels maintained within the upper limit of the normal range for each sex. Androstenedione level was measured by a commercially available assay (Elecsys Androstenedione immunoassay kit (Roche Diagnostics).

Pubertal onset was defined as breast Tanner stage 2 in girls and a testicular volume of 4 ml in boys. Precocious puberty was defined as the appearance of 2ry sexual characteristics before the age of 8 years in girls and before 9 years in boys. Central precocious puberty was confirmed by pubertal level of LH after formal gonadotropin-releasing hormone (GnRH) stimulation test >5 IU/L, whereas peripheral precocious puberty (PPP) was documented when LH levels remained within the pre-pubertal range<5 IU/L (7).

Primary amenorrhea was defined as the absence of menarche at the age of 15 years or thereafter in the presence of normal growth and secondary sexual characteristics. Secondary amenorrhea was defined as the absence of menses for more than 3 months in girls or women who previously had a regular menstrual cycle or six months in those who had irregular menses. Oligomenorrhea was defined as having fewer than nine menstrual cycles per year or a cycle length exceeding 35 days (23). Hirsutism was clinically evaluated, but no standardized scoring system was documented in the patients’ medical records.

Bone mineral density (BMD) was assessed by dual x-ray absorptiometry performed within the last 2 years. Osteoporosis was defined as a T-score of lower than or equal −2.5 SD, while osteopenia was defined as a T-score between −1 and −2.5 SD at the femoral neck in patients more than 25 years of age. While in patients less than 25 years of age at the time of study, Z-score was used to evaluate the bone density as low BMD was defined as a z-score of -2SD or less at the lumbar spine and/or whole body (22).

The patients underwent a metabolic evaluation after a 12-hour fast, including lipid profile (LDL, HDL, triglycerides, and total cholesterol), fasting glucose, HbA1c, and an oral glucose tolerance test (OGTT). Diagnosis of diabetes and prediabetes followed the American Diabetes Association (ADA) criteria (24). We defined dyslipidemia according to established guidelines, primarily NCEP ATP III and AHA/ACC criteria (25, 26).

### 2.2 Genetic testing

Genetic testing was conducted as part of routine practice. DNA libraries were prepared using the Agilent Sureselect All Exons V6 (50 Mb) capture kit and sequenced on an Illumina HiSeq2500 platform with an average target depth of 80×. The exome data were aligned to the human reference genome (NCBI build 37.1, UCSC hg19) and analyzed using the QIAGEN Clinical Insight (QCI) Interpret, which includes copy number variation detection. In addition, an in-house variant interpretation pipeline was utilized, incorporating databases of known disease-causing variants in the Saudi population and aggregated variants from the Center for Genomic Medicine (CGM-DB).

### 2.3 Statistical analysis

Descriptive analysis was performed using STATA software version 17 for Windows. Continuous data were reported using the mean, standard deviation (SD), median, and interquartile ranges [IQR]. Categorical data were presented in frequencies (n), percentages (%), and bar graphs.

### 2.4 Ethical considerations

The study was approved by the Ethics Committee of King Faisal Specialist Hospital and Research Centre (reference: 2245527). The studies were conducted in accordance with the local legislation and institutional requirements. The ethics committee/institutional review board waived the requirement of written informed consent for participation from the participants or the participants’ legal guardians/next of kin because of the retrospective nature of the study.

## 3 Results

### 3.1 Patients

A total of 108 patients with 21-hydroxylase deficiency were included in this study. Most of the patients were female (66.67%), whereas males represented only one-third of the patient cohort (33.33%). The median age of the patients was 21 years, with an IQR of 18–30. Of the studied patients, 93.51% had the classic form of 21-hydroxylase deficiency (Salt-wasting CAH), while only 6.49% had the simple virilizing form. All female patients presented with ambiguous genitalia, leading to a referral to our hospital for genital reconstruction early in infancy. All 46 XX patients were raised as females, except one patient who had severe virilization and underwent hysterectomy and ovarian removal and was raised as a male. The hormonal control was variable among our patients. Androstenedione levels were normal in 51.85% and were persistently elevated in 48.15% of the patients with mean androstenedione levels of 17.04 nmol/l and 14.1 nmol/l for males and females, respectively. In total, 9.25% of the patients were prehypertensive, while 3.71% were diagnosed with hypertension and followed on antihypertensive medications. Three patients were on angiotensin converting enzyme inhibitors (ACEIs) enalapril 5 mg, 10 mg, and 20 mg per day respectively and one patient on calcium channel blocker CCBs nifedipine 20 mg per day. All hypertensive patients had the classic form of CAH, except for one patient with a simple virilizing form. Puberty onset was normal in 69.14% of the participants, precocious in 22.22%, and delayed in 8.64%. Hirsutism was reported in 30% of the female patients, and 12.96% of the participants had acne (**Table 1**).

**Table 1:** Clinical characteristics of the study patients.

Characteristics		n (%)	Total
Gender	-Males	36 (33.33)	108
	-Females	72 (66.67)	
Age (in years)	Median [IQR]	21 [18–30]	108
Diagnosis	-Salt-wasting	101 (93.51)	108
	-Simple virilizing	7 (6.49)	
Ambiguous genitalia	-Yes (all females)	72 (66.67)	108
	-No (all males)	36 (33.33)	
Height	Height SD-score: mean ± SD	−2.22 ± 1.37	108
	-Short (SD-score <−2)	31 (28.70)	
	-Severe short (SD-score <−3)	33 (30.55)	
	-Normal	44 (40.74)	
BMI	-Underweight	9 (8.33)	108
	-Normal	38 35.19)	
	-Overweight	23 (21.30)	
	-Obese	38 (35.19)	
Blood pressure	-Normal	94 (87.04)	108
	-Pre-hypertensive	10 (9.25)	
	-Hypertensive	4 (3.71)	
Puberty onset*	-Precocious	18 (22.22)	81
	-Normal	56 (69.14)	
	-Delayed	7 (8.64)	
GnRH agonist	Yes	18 (16.6)	108
	-Height SD-score for patients received GnRH agonist	-2.47 ± 1.50	
	-Height SD-score for patients without GnRH agonist	-2.18 ± 1.36	
Acne	-Yes	14 (12.96)	108
	-No	94 (87.04)	
Female complications and treatment			
Hirsutism	-Yes	22 (30.56)	72
	-No	50 (69.44)	
Menstrual	-Primary amenorrhea	12 (16.67)	72
	-Secondary amenorrhea	10 (13.89)	
	-Oligomenorrhea	42 (58.33)	
	-Normal menses	8 (11.11)	
Fertility treatments**	-IVF	1 (1.38)	25
	-None	24 (33.34)	
On Contraceptive pills*	Yes, type:	22 (30.56)	72
	-Combined oral contraceptives	18 (81.82)	
	-Conjugated estrogen	1 (4.55)	
	-Progestin pills	3 (13.64)	

SD-score, Standard deviation score; BMI, Body mass index; IQR, Interquartile range; IVF, In vitro fertilization.

*Only patients that we found data on were reported.

**Only 25 females are married.

### 3.2 Steroids replacement therapy

As listed in **Table 2**, almost half of the patients were on prednisone (46.30%) with a mean dose of 5.78 mg per day, followed by hydrocortisone (37.97%) with a mean dose of 21.53 mg and dexamethasone (12.03%) with a mean of 0.4 mg. Furthermore, 2.78% of the patients were on hydrocortisone and dexamethasone combination, and only one patient (0.92%) was followed on prednisone and dexamethasone combination. Most of the classic form patients (97%) were on fludrocortisone replacement therapy with a mean dose of 0.09 mg per day. The mean current glucocorticoid dose is 23 mg.

**Table 2:** Steroid replacement therapy by dose.

Current replacement therapy	n (%)	Mean dose (mg) (range)
Fludrocortisone (in salt-wasting cases)	98/101 (97.03)	0.09 (0.05–0.1)
Dexamethasone	13/108 (12.03)	0.4 (0.25–0.5)
Hydrocortisone	41/108 (37.97)	21.53 (7.5–30)
Prednisone	50/108 (46.30)	5.78 (2.5–10)
Overall current glucocorticoid dose		23 (6.7–40)
Glucocorticoid Combinations		
Hydrocortisone and Dexamethasone	3/108 (2.78)	15, 0.25*
Prednisone and Dexamethasone	1/108 (0.92)	2.5, 0.5**

*All three patients received identical doses of hydrocortisone (15 mg) and dexamethasone (0.25 mg).

**One patient received doses of prednisone (2.5 mg) and dexamethasone (0.5 mg).

Notably, one of the female patients with classic CAH underwent bilateral adrenalectomy at the age of 18 due to severe uncontrolled virilization, and she is currently on prednisolone 5 mg per day and fludrocortisone 0.1 mg daily with no complications.

### 3.3 Growth data

The mean height standard deviation score (SDS) for our patients was -2.22. Among them, 40% had normal height (mean SDS: -0.97), 28% were classified as short (mean SDS: -2.39), and 30% were considered severely short (mean SDS: -3.74). Notably, all severely short patients had the classic form of CAH, except for one female patient with the simple virilizing form, who presented with precocious puberty. Additionally, patients who received GnRH analogs had a mean height SDS of -2.47, compared to -2.18 in those who did not receive treatment (**Table 1**).

As for obesity, 35.19% of the patient cohort were obese, 21.30% were overweight, 8.33% were underweight, and 33.33% had a normal BMI (**Table 1**).

### 3.4 Female reproductive health

Over 50% of the female patients had oligomenorrhea (58.33%), followed by primary amenorrhea (16.67%) and secondary amenorrhea (13.89%); only 11.11% of the patients had regular menses. At the time of the study, 30.56% of the female patients were taking oral contraceptive pills. Most of them (81.8%) were on combined oral contraceptives and 13% were on progestin-only pills. Furthermore, three females were married with children. All of them had spontaneous pregnancies, except one who used IVF treatment due to recurrent miscarriage (**Table 1**). Gonadal imaging (ultrasound) performed on 41 female patients revealed that 2.44% had polycystic ovaries, 12.20% had a small infantile uterus, and 85.36% had unremarkable findings. Ovarian rest tumors were unreported in our patients. Most of our female patients had normal FSH and LH levels with a mean value of 5.33 IU/L and 5.83 IU/L, respectively (**Table 3**).

**Table 3:** Laboratory findings.

Lab requested (unit)	n (%), mean ± SD
Cholesterol (mmol/l)	4.50 ± 0.92
-Normal <5.17	84 (77.78)
-Borderline 5.17–6.20	19 (17.59)
-High ≥ 6.21	5 (4.63)
LDL (mmol/l)	2.87 ± 1.20
-Optimal < 2.59	46 (42.59)
-Low risk 2.59–3.34	39 (36.11)
-Borderline 3.37–4.12	16 (14.81)
-High ≥ 4.14	7 (6.48)
HDL (mmol/l)	1.46 ± 0.41
-Low <1.3	31 (28.70)
-Desirable ≥ 1.3	77 (71.30)
Triglyceride (mmol/l)	1.12 ± 0.55
-Desirable < 1.7	93 (87.74)
-Borderline 1.7–2.2	10 (9.43)
-High ≥ 2.3	3 (2.83)
HbA1c (%)	5.40 ± 0.64
-Normal < 5.7%	85 (78.70)
-Prediabetes 5.7–6.4%	20 (18.52)
-Diabetic ≥ 6.5%	3 (2.78)
2-Hour Postprandial Glucose (mmol/L)	5.71 ± 1.06
-Normal <7.8	106 (98.15)
-Impaired Glucose Tolerance 7.8–11.0	1 (0.93)
-Diabetes ≥ 11.1	1 (0.93)
Glucose fasting mmol/l	4.71 ± 0.58
-Normal < 5.6	100 (92.59)
-Impaired 5.6–6.9	7 (6.48)
-Diabetic ≥7.0	1 (0.93)
HOMA-IR	2.00 ± 1.78
-Healthy ≤ 2.0	67 (62.04)
-Insulin resistance > 2	41 (37.96)
Androstenedione (nmol/L)*	
-Mean level in males	17.04 ± 14.66
-Mean level in females	14.10 ± 15.02
Category:	
-Controlled	56 (51.85)
-Persistently high	52 (48.15)
Testosterone (nmol/L)**	
-Mean level in males	11.88 ± 7.95
-Mean level in females	3.29 ± 4.60
Category:	
-Low in males	19/36 (52.78)
-Persistently high in females	28/72 (38.89)
FSH (IU/L)	5.03 ± 5.22
-In males	4.43 ± 4.42
-In females	5.33 ± 5.59
Category:	
-Low <2.5	22 (20.37)
-Normal 2.5–12	84 (77.78)
-High >12	2 (1.85)
LH (IU/L), overall	5.37 ± 4.80
In males	4.46 ± 3.97
In females	5.83 ± 5.13
Category:	
-Low <2.5	23 (21.30)
-Normal 2.5–12	79 (73.15)
-High >12	6 (5.56)

HOMA-IR, Homeostatic Model Assessment of Insulin Resistance; FSH, Follicle-Stimulating Hormone; HbA1c, Glycated Hemoglobin; LDL, Low-Density Lipoprotein; LH, Luteinizing Hormone; SD, Standard Deviation.

*References range: for females: 2.27–7.33 nmol/L (65–210 ng/dL), for males: 2.79–8.38 nmol/L (80–240 ng/dL).

**Reference range: 0.5-2.5 nmol/L for females, and 10–35 nmol/L for males.

### 3.5 Male reproductive health

Testicular adrenal rest tumors (TARTs) investigated with ultrasound were observed in 12 out of 27 patients (44.44%) with mean number of reported lesions 2.0± 0.7 in the right side and 1± 0.7 in the left side. All lesions were reported as bilateral hypoechoic lesions with mean lesion size of 1.67 x 1.67 x1.38 cm in the right side and 1.72 x1.43x 1.13 cm in the left side. An additional male patient (3.70%) had previously undergone partial orchidectomy, with histopathology reporting a benign Leydig cell tumor, and the remaining 14 patients (51.86%) had an unremarkable gonadal ultrasound (**Table 4**). The youngest patient with TARTs was 16 years old. Most of our male patients had normal FSH and LH levels with a mean value of 4.43 IU/L and 4.46 IU/L, respectively (**Table 3**). In addition, hypogonadotropic hypogonadism was diagnosed on a hormonal basis in one male patient with classic CAH who was on testosterone replacement therapy at the time of the study. Another patient was diagnosed with Klinefelter syndrome (47xxy), and he was experiencing primary gonadal failure, for which he was receiving replacement therapy as well.

**Table 4:** Radiological findings.

Radiology findings	n (%)
Bone mineral density n=60	
T score (25 years old and Above) (n=23)	-1.32 ± 1.14
-Normal (t-score +1 and −1)	7 (30.43)
-Osteopenia (t-score −1.1 to −2.4)	13 (56.52)
-Osteoporosis (t-score ≤ −2.5)	3 (13.04)
Z score (Below 25 years old) (n=37)	-0.88 ± 1.35
-Normal (z-score >-2)	29 (78.38)
-Low for age (z-score ≤−2.0)	8 (21.62)
Gonadal ultrasound findings	
For males n=27	
Bilateral TART	12 (44.44)
Leydig cell tumor	1 (3.70)
Unremarkable	14 (51.86)
For females: n=41	
Polycystic ovaries (PCO)	1 (2.44)
Small uterus	5 (12.20)
Unremarkable	35 (85.36)

TART, Testicular adrenal rest tumor.

### 3.6 Metabolic abnormalities

For metabolic abnormalities, cholesterol levels were high in most of the patients (95.65%) with a mean of 4.4 mmol/l. LDL was elevated in 9.53% with a mean of 2.87 mmol/l, and triglyceride levels were normal across the whole patient cohort. None of our patients were started on statin therapy. The majority had normal HBA1C (78.67%) with a mean of 5.39%, but 17.33% were prediabetic, and 4% were classified as diabetic. Three patients were on metformin, and one patient was on metformin plus an oral hypoglycemic drug (**Table 3**).

### 3.7 Bone mineral density

Among the 60 patients assessed for BMD, 23 were 25 years or older, and their T-scores indicated that 30.4% had normal BMD, 56.5% had osteopenia, and 13.0% had osteoporosis. For the 37 patients under 25 years old, the Z-score analysis showed that 78.4% had normal bone density, while 21.6% had bone density classified as low for age (**Table 4**).

### 3.8 Genetic test

The genetic test was done in 55 patients, and all of them had a positive *CYP21A2* gene. The most reported variants were *c.293-13C>G* (n = 23), c.955C*>T* (n = 15), and *c.952C>T* (n = 12) (**Table 5**).

**Table 5:** *CYP21A2* gene mutations (transcript: NM_000500.9) n = 55:.

Exon	Change at the DNA level	Change at the protein level	Type	ACMG classification	dbSNP	n*
N/A	30-KB DEL	N/A	Gene deletion	P	N/A	6
1	c.92C>T	p. Pro31Leu	SNV	P	rs9378251	2
Intron 2	c.293-13C>G	N/A	SNV	LP	rs6467	23
3	c.332_339del	p. Gly111ValfsTer21	Variants deletion	P	rs387906510	2
4	c.518T>A	p. Ile173Asn	SNV	P	rs6475	1
6	c.710_719del	p. Ile237SerfsTer3	Variants deletion	LP	N/A	1
6	[c.710T>A, c.713T>A, c.719T>A]	p. Ile237Asn, p. Val238Glu, p. Met240Lys	SNV Cluster	LP LP VUS	rs1554299737 rs12530380 rs6476	3
7	c.803C>G	p. Pro268Arg	SNV	VUS	rs61732108	1
7	c.844G>T	p. Val282Leu	SNV	P	rs6471	1
7	c.923_924dup	p. Leu309CysfsTer15	Variant duplication	P	rs1776193326	1
8	c.952C>T	p. Leu318=	SNV	P	rs1776217797	12
8	c.955C>T	p. Gln319Ter	SNV	P	rs7755898	15
8	c.1066C>T	p. Leu356=	SNV	P	rs1776234662	2
8	c.1069C>T	p. Arg357Trp	SNV	P	rs7769409	1
9	c.1131C>G	p. Tyr377Ter	SNV	LP	N/A	1
10	c.1478A>G	p. Gln493Arg	SNV	VUS	N/A	1

N/A, not applicable; P, Pathogenic; LP, Likely Pathogenic; VUS, Variant of Uncertain Significance; SNV, single nucleotide variant; N, number of cases within the variant; ACMG, American College of Medical Genetics and Genomics.

*Of the 55 patients who underwent genetic tests, 47 had single mutations, while 8 had compound mutations (2 mutations: n=2; 3 mutations: n=3; 4 mutations: n=2; 5 mutations: n=1).

## 4 Discussion

In our study, we reported the long-term health outcomes of CAH due to 21-hydroxylase deficiency in adolescent and adult patients who were enrolled in a single tertiary institution in Saudi Arabia.

Our data demonstrated reduced adult height for more than half of the patients compared with the general population. Overweight and obesity were the most commonly reported outcomes. Most female patients had menstrual complications that impacted their menstrual cycles, such that only 11.11% had regular menses. The administration of oral contraceptive pills was noted in one-third of our female patients. For male patients who underwent ultrasound investigation (27 patients), 44.44% had TARTs.

Our analysis revealed that more than half of our patients (59.5%) had reduced final adult height, corroborating previously reported international findings (7, 22, 27). Also, in alignment with a recent Saudi study reported short stature as the most common complication (67%) in which the mean adult height for males and females 156 cm and 147 cm, respectively (6). A systematic review and meta-analysis of 35 studies regarding adult height in CAH concluded that the final height of patients with CAH on glucocorticoid treatment was lower than that of the general population and lower than their genetic height potential (28). Recent studies, however, have reported improved height outcomes in patients with CAH, which could indicate better therapeutic strategies and adequate patient follow-up (28, 29). Reduced final adult height can be attributed to both undertreatment with glucocorticoids and prolonged exposure to non-physiological doses. These factors directly suppress the growth plate and affect the growth hormone-insulin-like growth factor 1 axis, leading to the same result (8, 29, 30). Recent reports show that final adult height was worse in patients treated with glucocorticoids alone compared to those receiving both glucocorticoids and mineralocorticoids (29)—an observation that could not be tested in our study, as more than 90% of our patients were diagnosed with classic salt-wasting CAH and received mineralocorticoids.

Some authors have suggested that high glucocorticoid doses during infancy contribute negatively to the final adult height, especially since the available formulation of hydrocortisone replacement therapy used in most of the world does not offer the lowest recommended physiological doses during that period (9, 31). Growth-promoting agents such as growth hormone therapy with or without GnRH analog have been recommended to improve the final adult height only in CAH patients with markedly short predicted adult height, although prospective randomized controlled studies are still needed to determine their efficacy to preserve the final height (17). In our study, central precocious puberty (CPP) was documented in approximately one-fourth of the patients, all of whom were treated with GnRH analogs. We found discrepancy in growth outcomes between those who received GnRH analogs and those who did not which may be attributed to either the late initiation of GnRH therapy or the presence of significant hyperandrogenism, which, in turn, necessitated non-physiological steroid dosing, potentially affecting growth trajectories.

Data derived from recent literature indicate that the risk factors for cardiometabolic complications are common in adults with CAH compared with the normal population, including obesity, insulin resistance, dyslipidemia, and hypertension (2, 8). A recent systematic analysis, which included 20 studies evaluating cardiovascular and metabolic risk in patients with CAH compared with matched controls, concluded that the former had higher blood pressure, insulin resistance, and carotid intima thickness. Furthermore, Falhammar et al. reported increased mortality among patients with CAH cardiovascular events as the second most common cause of death after adrenal crisis (32). Obesity is a main risk factor for cardiometabolic morbidities in both pediatrics and adults (10). In our cohort, over half of the patients were either overweight or obese due to prolonged exposure to high glucocorticoid doses. This finding aligns with previous reports from a large cohort, where nearly one-third of pediatric and adult patients were obese (22). Also, aligns with the recent Saudi study where more than one-third of their included patients were obese (6). Additionally, a study examining body composition in patients with CAH found that both visceral and abdominal adipose tissue were increased in these patients, leading to insulin resistance (33). Although insulin resistance is commonly observed in patients with CAH and is typically evaluated using HOMA, we assessed glucose homeostasis using HbA1c. Prediabetes was documented in only a small number of patients compared to other reported studies (10), and just three patients in our cohort were diagnosed with diabetes. Similarly, we found that most of our patients had acceptable lipid profiles and that none of them were started on statins. The observed lower prevalence of dyslipidemia and diabetes mellitus in our study was because most of our patients were younger than 40 years of age, which corroborates previous studies reporting the worsening of metabolic outcomes in patients with CAH, aged over 40 years (3, 34).

Hypertension in patients with CAH could result from iatrogenic hypercortisolism beyond mineralocorticoid overexposure. Obesity alone can contribute to hypertension development in these patients, although it is not consistent in all previous studies (2, 32). In our study, most of our patients had normal blood pressure, except four patients found to be hypertensive and controlled on antihypertensive medications. All four patients were obese, except one who had a normal BMI. The correlation between blood pressure and BMI or fludrocortisone doses was not well established in our study. The low prevalence of hypertension in our study was similar to that in a recent German cohort (35), but contradicted the results of an American cohort, as hypertension was reported in two-thirds of their classic CAH patients (22).

Reproductive outcomes have been extensively studied among adults with CAH. As a consequence of CAH and its treatment, both males and females suffer from reduced fertility but exhibit different manifestations. In females, non-physiological doses of steroids can result in hypogonadotropic hypogonadism, which contributes to abnormal gonadal function and the resultant irregular menstrual cycle, anovulation, and low fertility rate (2). Furthermore, complications of genital reconstructive surgeries and various psychosocial factors that affect the desire to become pregnant have been reported as important contributing factors. Ovarian rest tumors have been reported but are less frequent than TARTs among females with CAH (2, 36). Overall, 16% of our female patients had primary amenorrhea despite being on oral contraceptive pills, which corroborates a previous cohort study conducted in the UK (27). Although menstrual cycle abnormalities were common in our cohort, it has been found that those abnormalities can also be common compared to matched control (37). The pregnancy rate in our cohort was very low, largely due to the low rate of marriage among adult females. However, consistent with other studies, those who attempted to conceive achieved successful pregnancies (27, 37). All females who conceived had the classic salt-wasting form. Hirsutism was observed in less than one-third of our female patients, a lower prevalence compared to other studies (27). This discrepancy may be due to incomplete data documentation. Moreover, the retrospective nature of the study precludes establishing a relationship between hirsutism and contraceptive pill use.

TART formation has been reported to be highly prevalent in males with CAH, with some studies indicating rates as high as 90% from puberty into adulthood. The prevalence can be even higher in those with poor hormonal control and persistently elevated ACTH levels, sometimes occurring before puberty. TARTs were also reported in patients with suppressed ACTH due to glucocorticoid overtreatment (10). Our study observed TARTs in 44% of 27 screened male patients. Similarly, a study by Finkielstain et al. in the US reported TART in classic males (33% boys, 44% men) (22). The rate of TART might have been higher if all the male in our study had been screened with ultrasound and if there had been more male with CAH in the study, since most of the patients were female. A higher prevalence of almost 70% of screened adult males has been reported for a population in the UK (27). One patient in our study underwent partial orchiectomy, with histopathological report revealing a benign Leydig cell tumor. Nonetheless, due to its resemblance to TART and the lack of a secondary review, the possibility of misdiagnosis persists, with TART being the more probable diagnosis. This highlights the significance of expert pathological assessment to prevent unnecessary surgical procedures. Hypogonadotropic hypogonadism was reported in only one male patient characterized by prepubertal testes and low levels of gonadotropins and testosterone, but a link to hyperandrogenism could not be established due to the retrospective nature of our study and some unreported data. Additionally, none of the male patients were married, so fertility could not be assessed.

Low BMD has been documented in adults with CAH, attributed to prolonged exposure to high glucocorticoid doses (22). Nevertheless, normal BMD has also been reported in another research (10). In our cohort, the majority had normal BMD, with only three patients having osteoporosis. All of them were females with the salt-wasting form and maintained on long-acting potent steroids (prednisolone and dexamethasone) known to have more adverse effects on bone health. We believe that they had prolonged steroid exposure, as indicated by their suppressed androgen level, short stature, and obesity. Notably, not all included patients underwent BMD examinations. Finkieelstain et al. reported a similar result, as 37% of their adult patients had low BMD (22) for which preventive measures for osteoporosis were suggested in that population. Furthermore, another Korean study (3) concluded that fracture risk increases with age in adults with CAH, especially those who exceed 40 years of age. However, fractures were not reported in our patients, likely due to their young age, as most were under 40 years old.

Unfortunately, optimal hormonal control remains elusive with the currently available glucocorticoid formulations and regimens. Evidence of overtreatment, indicated by suppressed androgen levels, and undertreatment, indicated by excess androgens, continues to be commonly observed across various cohorts worldwide. Consistent with previous studies (22, 27, 35), hormonal control among our patients was variable. The majority were treated with prednisolone, followed by hydrocortisone, while only a small number were treated with dexamethasone. Four patients were on combination therapy with two forms of glucocorticoid. Although clear guidelines for optimal glucocorticoid therapy in adult patients with CAH remain lacking, recent meta-analysis data indicate that despite being the most potent steroid for suppressing androgens, dexamethasone has the worst adverse effects on bone health and body mass index compared to both hydrocortisone and prednisolone (38). Dexamethasone has also been associated with a high risk of insulin resistance and should be avoided (3). It is important to note that both dexamethasone and prednisolone are not part of the standard glucocorticoid replacement therapy for CAH in childhood and should be reserved for selected cases after completion of growth (17).

To the best of our knowledge, no published data exist from Saudi Arabia or a broader Middle Eastern region on the long-term outcomes of 21-hydroxylase deficiency in adult patients despite the rising incidence of congenital adrenal hyperplasia in Saudi Arabia. This gap in knowledge highlights the need for focused research to better understand the progression, complications, and overall impact of the condition in our region. Our findings emphasize the importance of close, lifelong follow-up for these patients, not only during childhood but also throughout adulthood. Continuous care by expert adult endocrinologists and other related specialists is crucial, as these individuals remain at risk for various comorbidities that can impact their quality of life. In addition, awareness of the disease and its impact from childhood to adulthood among physicians should be encouraged. All patients with CAH need frequent monitoring of their auxological parameters, blood pressure, plasma renin activity, and fludrocortisone adjustment. Furthermore, screening for TART should begin in early childhood, not just in patients with poor hormonal control, as TART is often asymptomatic. Frequent and wise glucocorticoid replacement therapy adjustments are necessary to minimize possible complications. Finally, we emphasize the need for new therapeutic strategies, such as modified-release formulations, for better hormonal control and health outcomes (9).

Our study has certain limitations, including being based on a single tertiary center’s experience and the retrospective nature of the data analysis. Additionally, some patients were lost to follow-up, which may have affected the completeness of the findings. Consequently, the results should be interpreted with caution and cannot be generalized to the broader population. Further prospective multicenter studies involving larger patient cohorts are recommended.

In conclusion, this study highlights the long-term health outcomes of adolescents and adults with 21-hydroxylase deficiency at a single tertiary center in Saudi Arabia. The data show a high prevalence of short stature, obesity, and menstrual irregularities in females. TARTs are common in males. Although metabolic and bone health outcomes are generally favorable, the variability in hormonal control and its associated complications underscores the need for individualized glucocorticoid therapy. Continuous monitoring and improved treatment strategies are essential for optimizing the quality of life of patients with CAH.

## Data Availability

The data presented in this study are deposited in the ClinVar repository, accession numbers SCV005421020, SCV005421021, SCV005421022, SCV005421023, SCV005421024, SCV005421025, SCV005421026, SCV005421027, SCV005421028, SCV005421029, SCV005421030, and SCV005421031, and can be accessed at https://www.ncbi.nlm.nih.gov/clinvar/.
